# Supplementary material for: Gene expression dysregulation domains are not a specific feature of Down syndrome
Source: Nat Commun. 2019 Jun 6;10:2489. doi: 10.1038/s41467-019-10129-9 (PMC6554309; doi:10.1038/s41467-019-10129-9)
Supplement: Supplementary file 1 — Supplementary Information [file 41467_2019_10129_MOESM1_ESM.pdf]

Gene Expression Dysregulation Domains are not a Specific Feature of Down Syndrome

Ahlfors *et al*.

Supplementary Figures

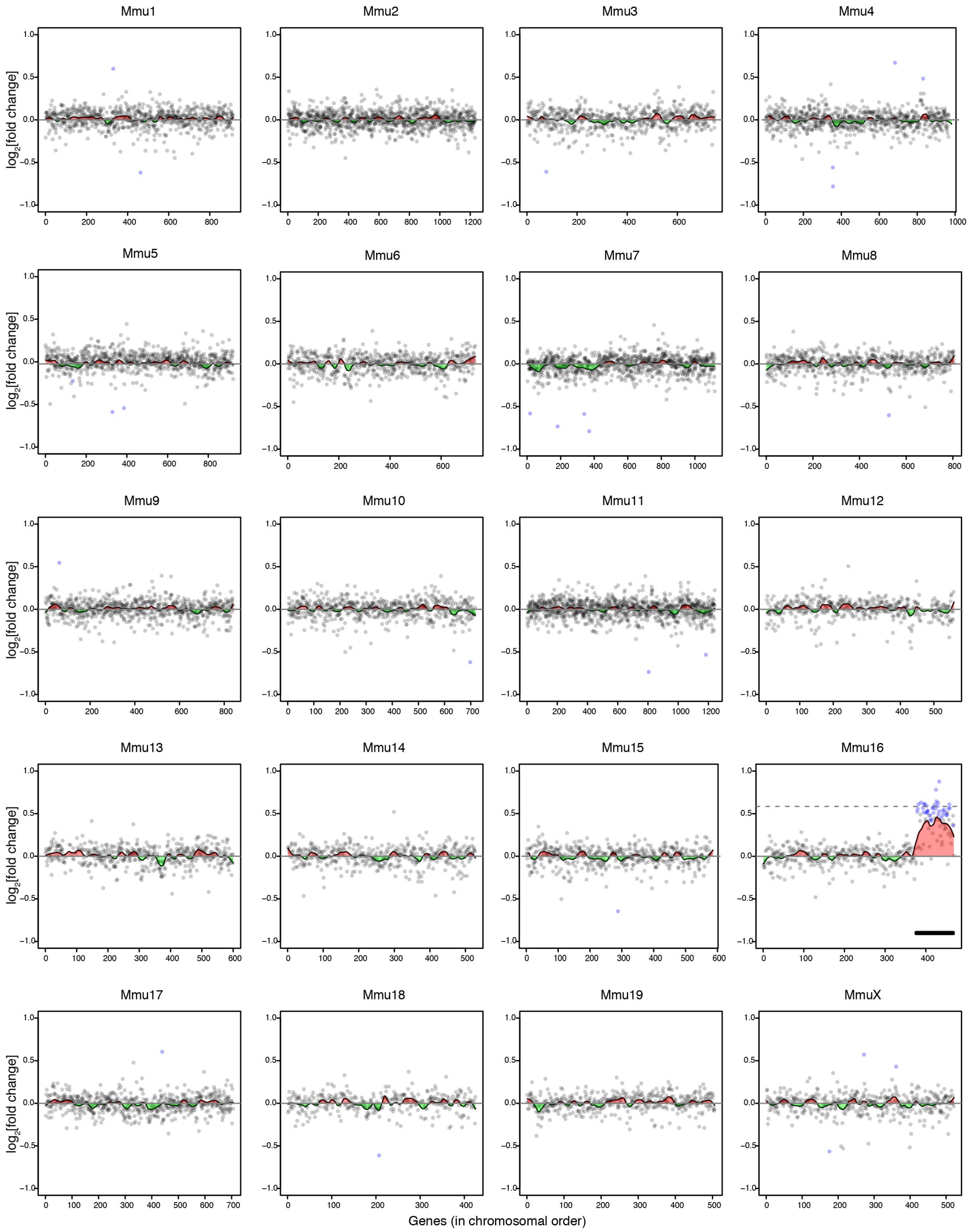

Supplementary Figure 1. Change in expression in Dp1Tyb MEFs as a function of chromosomal position. Plots show fold change of gene expression in Dp1Tyb MEFs v WT control cells. Expressed genes are plotted in chromosomal order. Genes that are significantly differentially expressed are indicated with blue dots, other genes are in grey. A Loess smoothing curve is superimposed with regions that are up- or down-regulated indicated in red or green. Dashed line on Mmu16 indicates a fold change of 1.5. Note the upregulation of genes in the duplicated region on Mmu16 (thick black line).

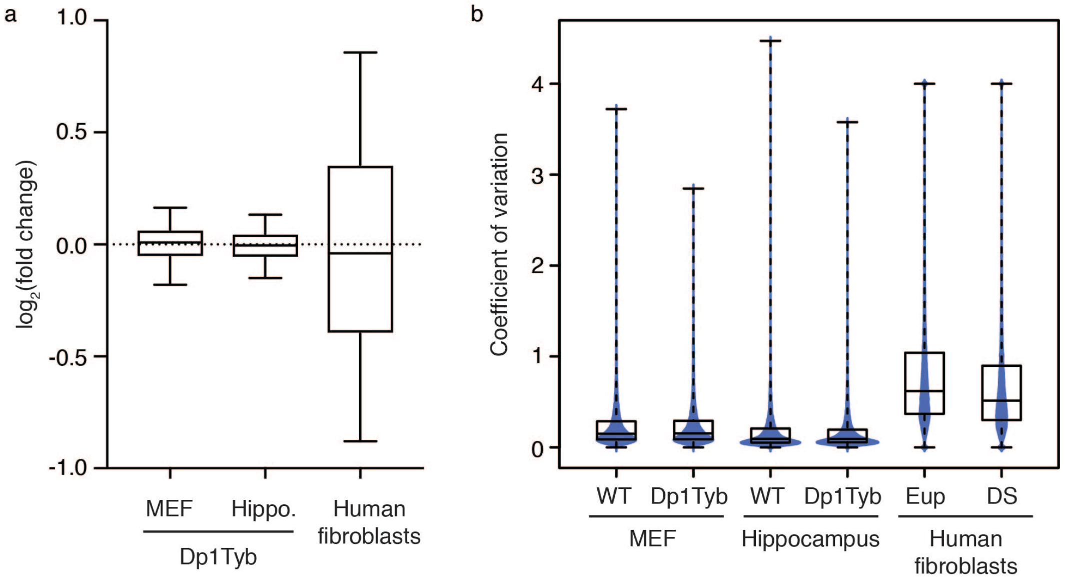

Supplementary Figure 2. Reduced gene expression changes and reduced variation in gene expression in Dp1Tyb MEFs and hippocampus compared to human DS fibroblasts. **a** Box and whisker plots of the distribution of fold changes in gene expression in the comparison of Dp1Tyb MEFs or hippocampus with WT controls, and in the comparison of DS and euploid human fibroblasts (data from Letourneau *et al*)[3]. Plots show median (black line), as well as the 25%-75% (box) and 5%-95% (whiskers) distributions. **b** Coefficient of variation (standard deviation/mean) of expression of every gene in RNAseq from WT and Dp1Tyb MEFs or hippocampus (this study) or euploid (Eup) and DS human fibroblasts (data from Letourneau *et al*)[3]. Plots show box (25%-75%) and whisker (0%-100%) plots superimposed on bean plots (blue) of the whole distribution.

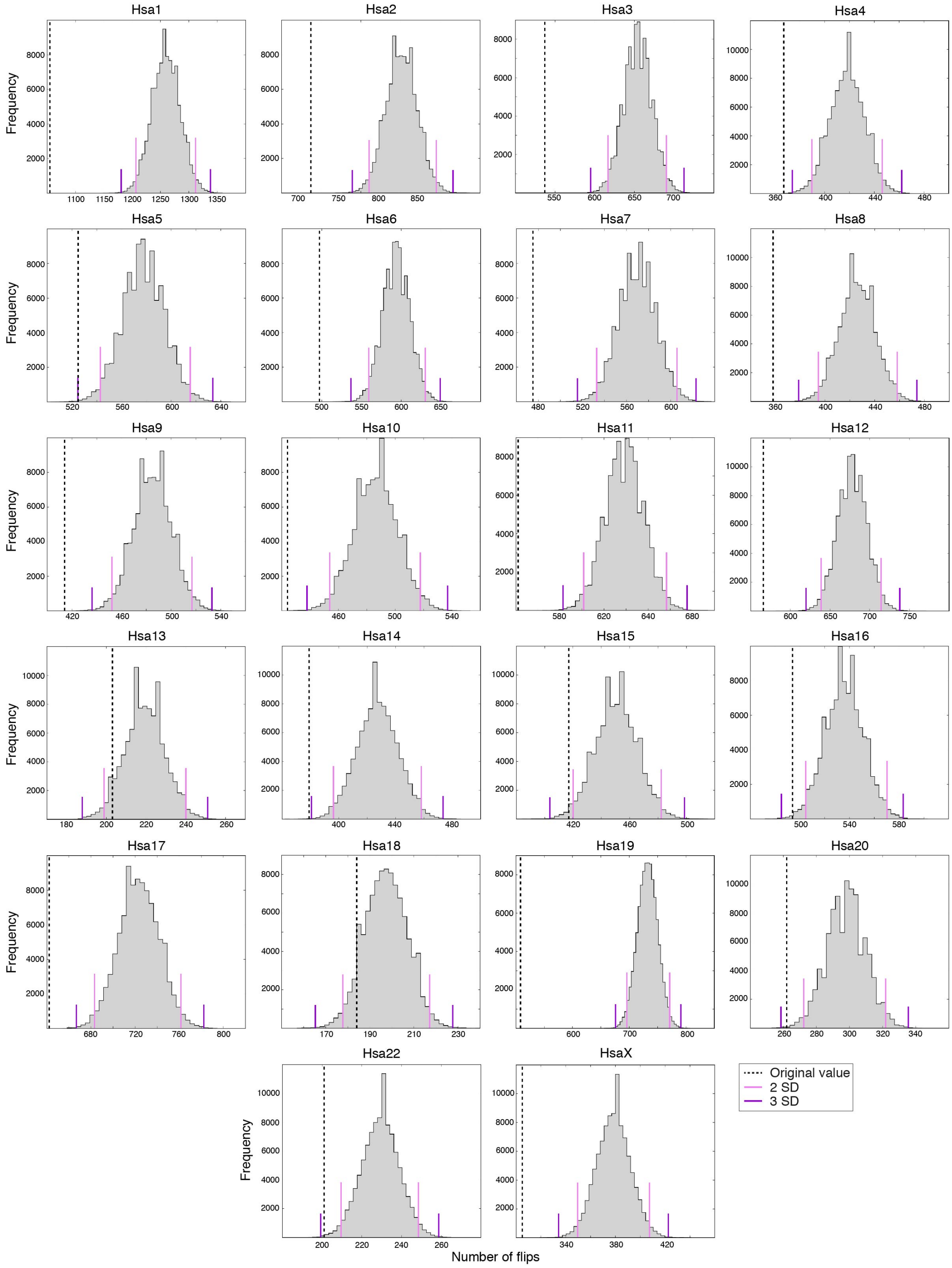

Supplementary Figure 3. Distribution of flips in gene expression in human DS fibroblasts[3]. Plots of distribution of numbers of flips in gene expression in 100,000 randomised versions of each chromosome in human DS v euploid fibroblasts. Pink and purple lines indicate 2 and 3 standard deviations (SD) away from the mean; dashed line shows the flips for the actual non-randomised chromosome.

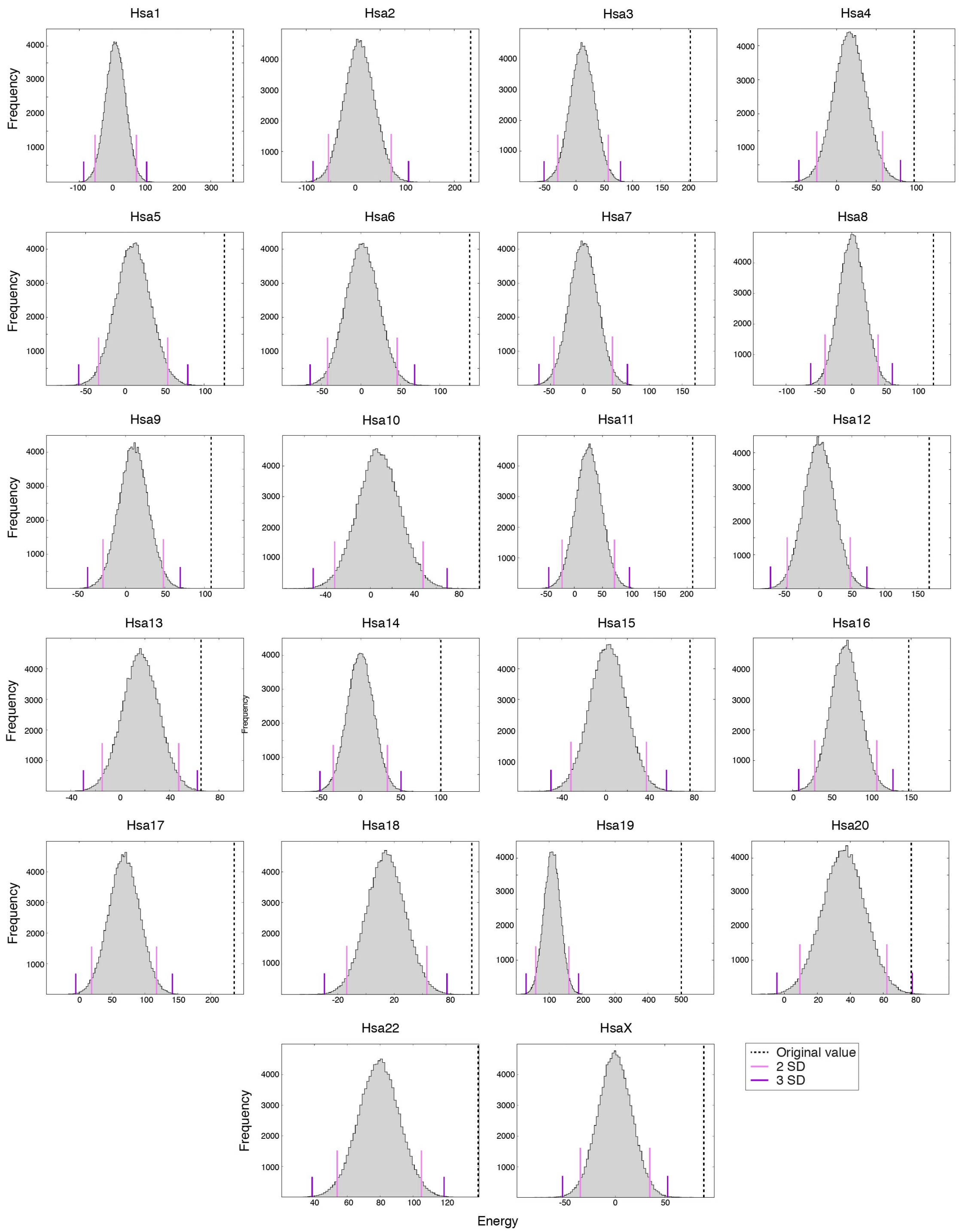

Supplementary Figure 4. Distribution of energy in gene expression in human DS fibroblasts[3]. Plots of distribution of energy in gene expression in 100,000 randomised versions of each chromosome in human DS v euploid fibroblasts. Pink and purple lines indicate 2 and 3 SD away from the mean; dashed line shows the energy for the actual non-randomised chromosome.

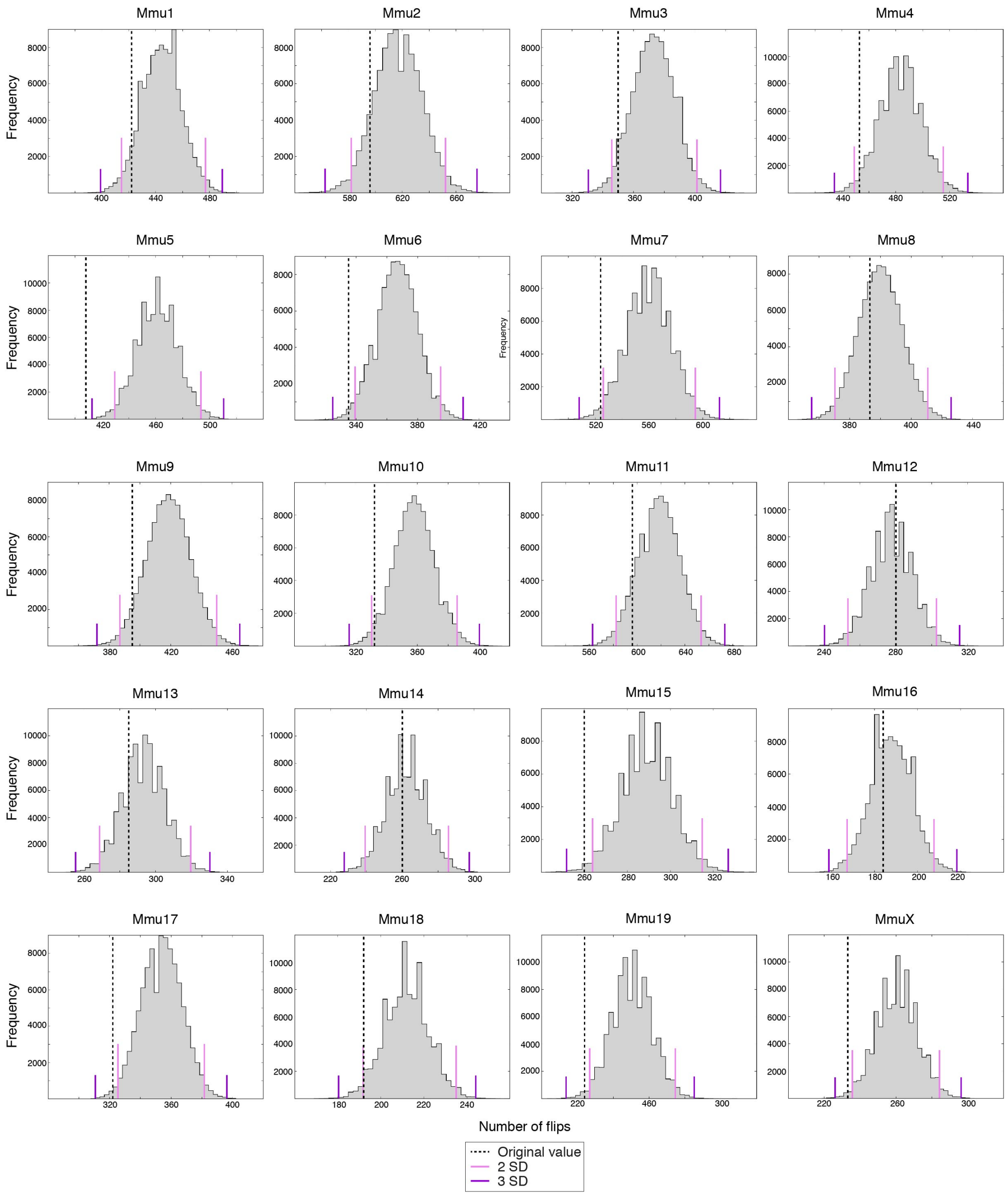

Supplementary Figure 5. Distribution of flips in gene expression in Dp1Tyb MEFs. Plots of distribution of numbers of flips in gene expression in 100,000 randomised versions of each chromosome in Dp1Tyb v WT MEFs. Pink and purple lines indicate 2 and 3 SD away from the mean; dashed line shows the flips for the actual non-randomised chromosome. Genes on Mmu16 that are duplicated in Dp1Tyb mice were excluded from this analysis.

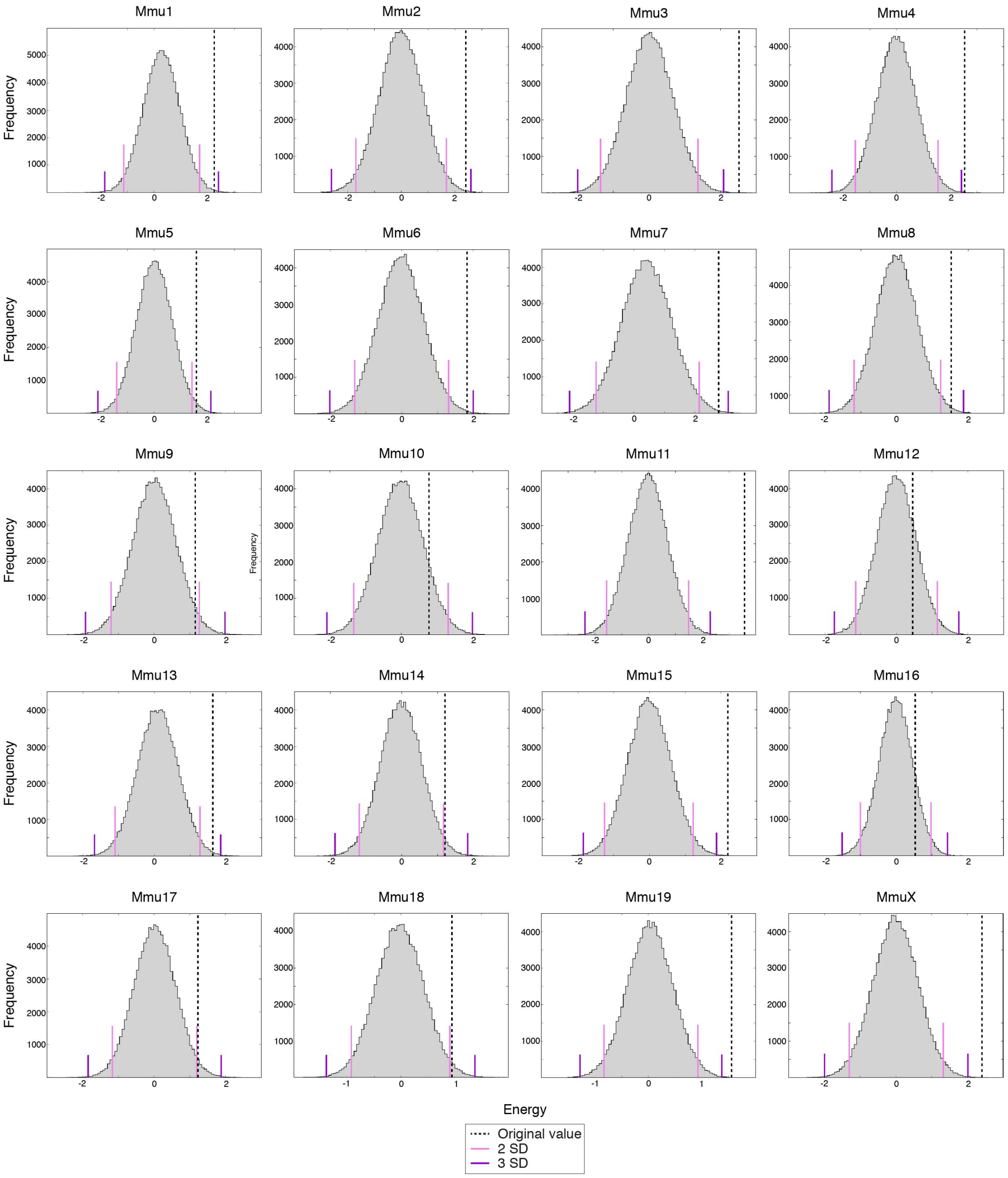

Supplementary Figure 6. Distribution of energy in gene expression in Dp1Tyb MEFs. Plots of distribution of energy in gene expression in 100,000 randomised versions of each chromosome in Dp1Tyb v WT MEFs. Pink and purple lines indicate 2 and 3 SD away from the mean; dashed line shows the energy for the actual non-randomised chromosome. Genes on Mmu16 that are duplicated in Dp1Tyb mice were excluded from this analysis.

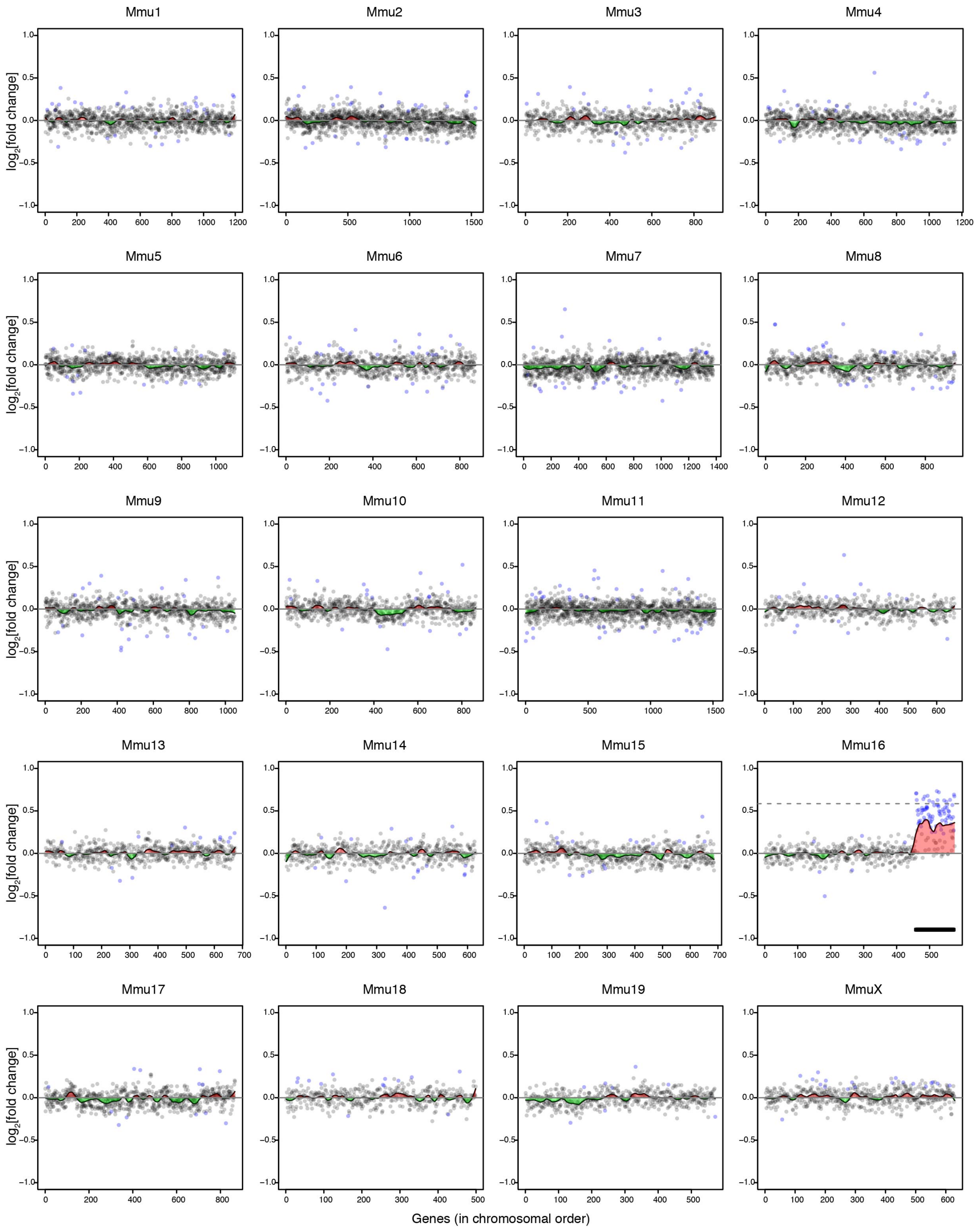

Supplementary Figure 7. Change in expression in Dp1Tyb hippocampus as a function of chromosomal position. Plots show fold change of gene expression in Dp1Tyb v WT hippocampus. Expressed genes are plotted in chromosomal order. Genes that are significantly differentially expressed are indicated with blue dots, other genes are in grey. A Loess smoothing curve is superimposed with regions that are up- or down-regulated indicated in red or green. Dashed line on Mmu16 indicates a 1.5 fold change. Note the upregulation of genes in the duplicated region on Mmu16 (thick black line).

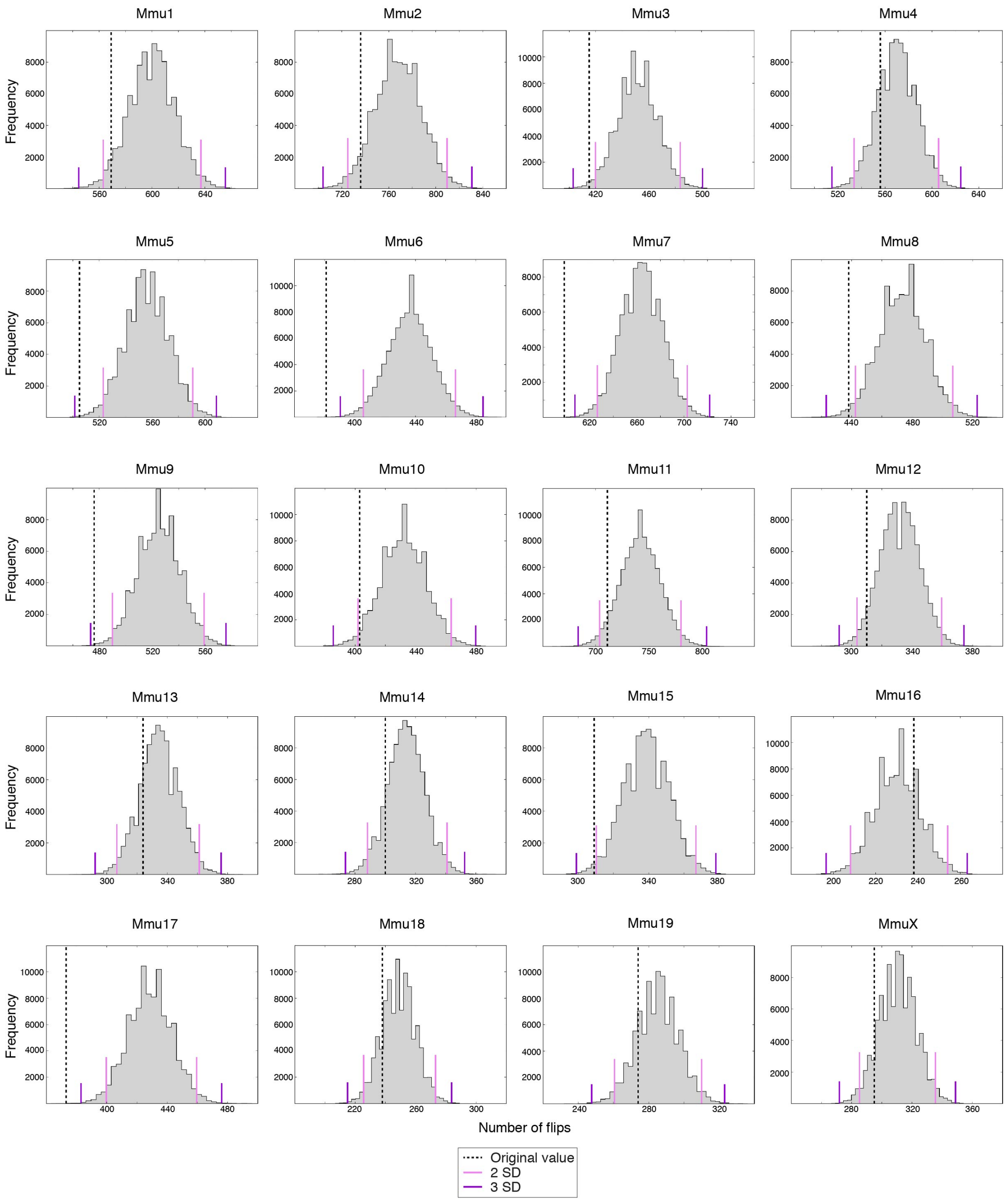

Supplementary Figure 8. Distribution of flips in gene expression in Dp1Tyb hippocampus. Plots of distribution of numbers of flips in gene expression in 100,000 randomised versions of each chromosome in Dp1Tyb v WT hippocampus. Pink and purple lines indicate 2 and 3 SD away from the mean; dashed line shows the flips for the actual non-randomised chromosome. Genes on Mmu16 that are duplicated in Dp1Tyb mice were excluded from this analysis.

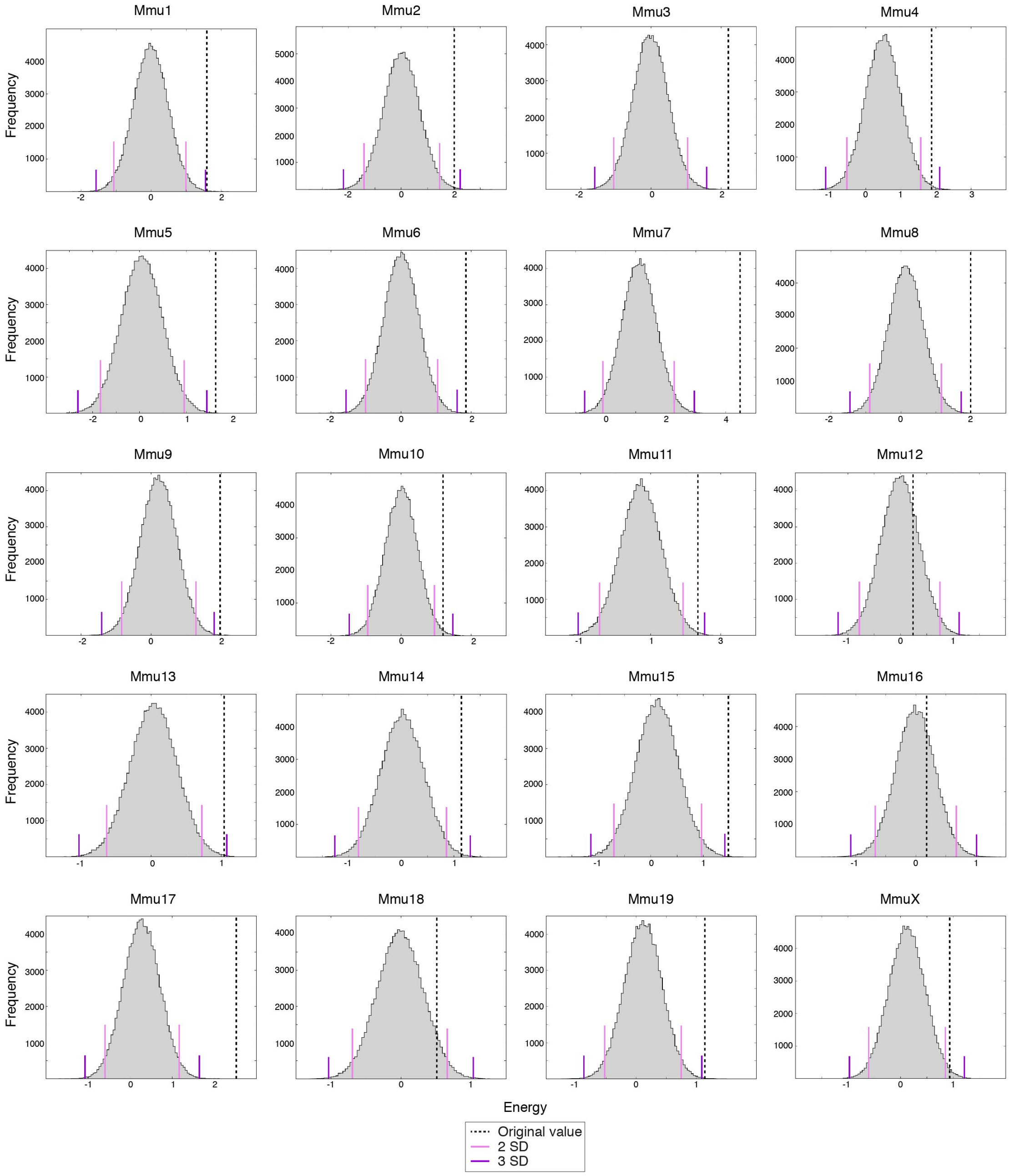

Supplementary Figure 9. Distribution of energy in gene expression in Dp1Tyb hippocampus. Plots of distribution of energy in gene expression in 100,000 randomised versions of each chromosome in Dp1Tyb v WT hippocampus. Pink and purple lines indicate 2 and 3 SD away from the mean; dashed line shows the energy for the actual non-randomised chromosome. Genes on Mmu16 that are duplicated in Dp1Tyb mice were excluded from this analysis.

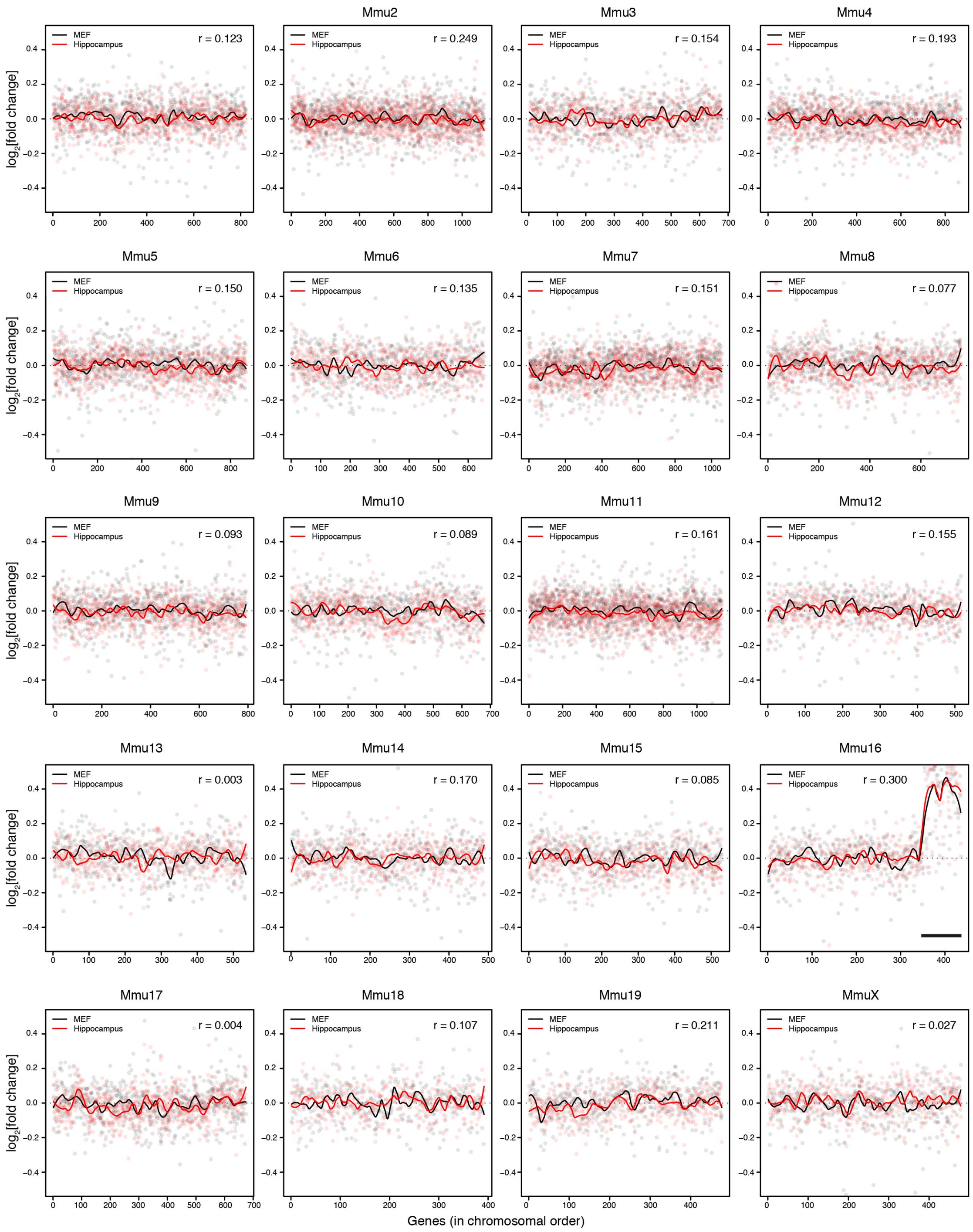

Supplementary Figure 10. Little correlation in gene expression changes between Dp1Tyb MEFs and hippocampus. Plots show fold change of gene expression in Dp1Tyb v WT MEFs and hippocampus. Expressed genes in MEFs (grey) and hippocampus (red) are plotted in chromosomal order. Loess smoothing curves for both MEFs (black) and hippocampus (red) are superimposed. The most obvious concordant gene expression changes are the upregulation of genes in the duplicated region on Mmu16 (thick black line). Correlation coefficient *r* is indicated for each chromosome. The duplicated region on Mmu16 was not included in the correlation calculation.

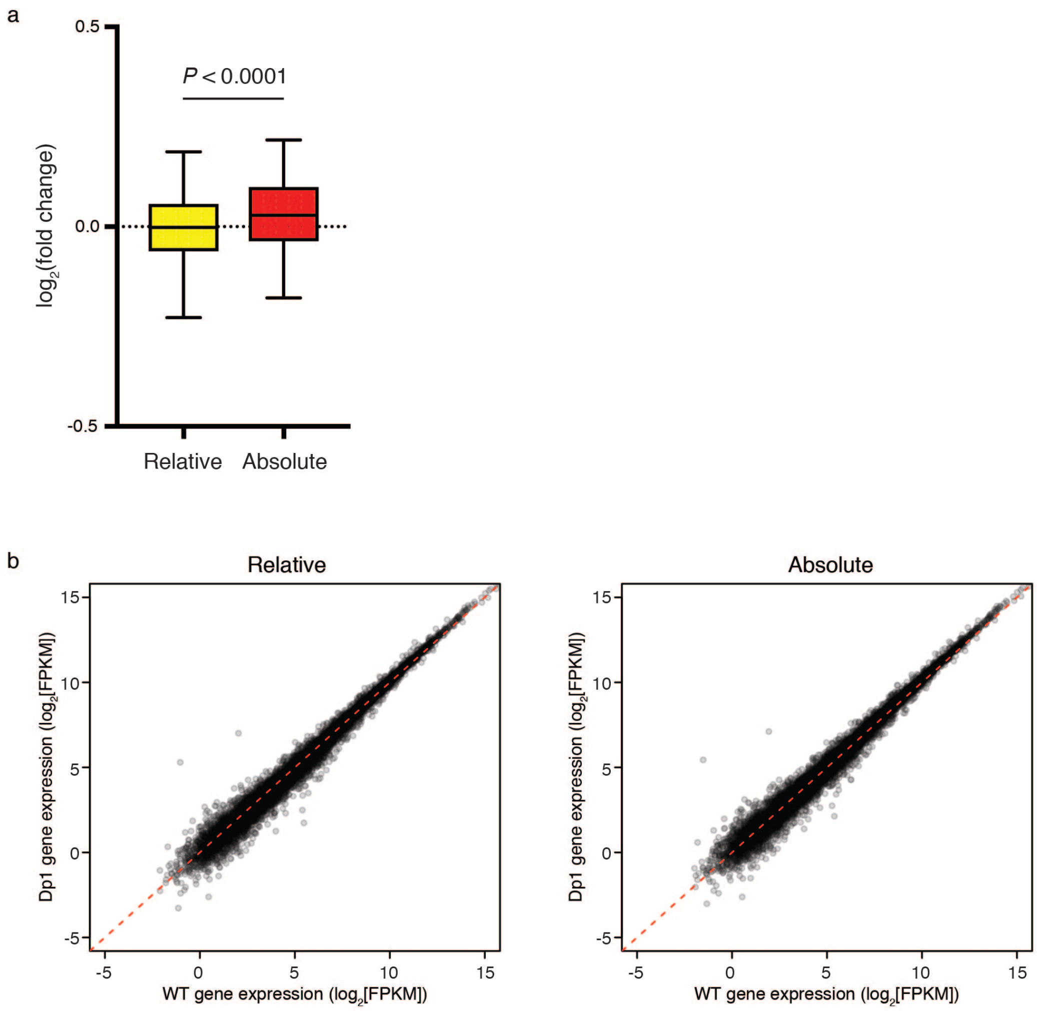

Supplementary Figure 11. Small increase in mRNA levels in Dp1Tyb MEFs compared to WT MEFs. **a** Box (25%-75%) and whiskers (5%-95%) plots of fold change in gene expression between Dp1Tyb and WT MEFs quantitated using either a relative normalisation to median gene expression or an absolute normalisation to spike-in control RNAs. A ~1.8% increase of fold changes in the absolute normalisation compared to relative normalisation indicates a small increase in mRNA levels in Dp1Tyb MEFs compared to WT MEFs. **b** Plots of gene expression in Dp1Tyb and WT MEFs calculated using relative or absolute normalisation. Each dot is a single gene. No evidence of a preferential increase in RNA levels for lower expressed genes. Dashed red line indicates equivalent expression between genotypes; *P* value from unpaired t-test.

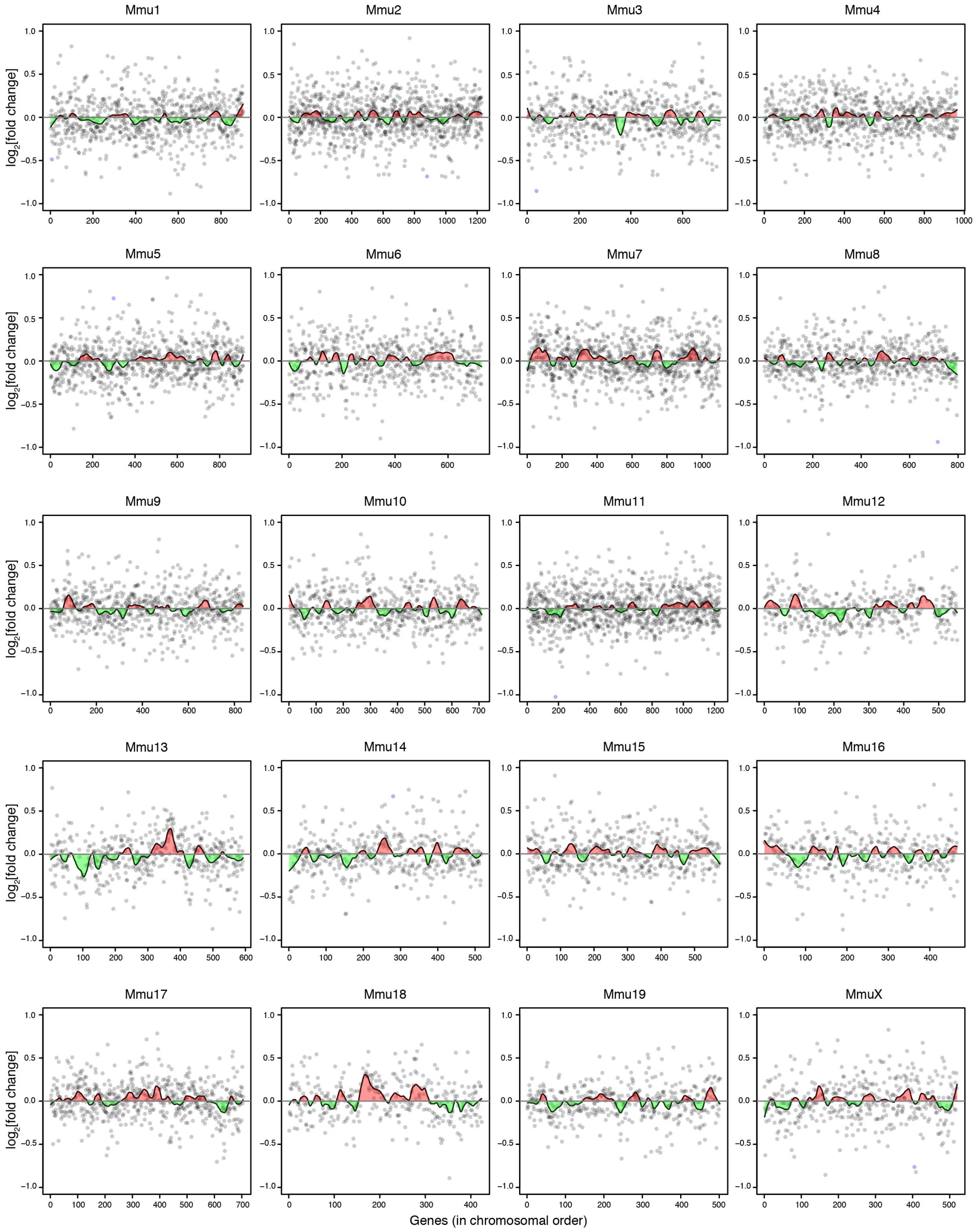

Supplementary Figure 12. Fold changes in gene expression from a no genotype difference comparison of 2 WT and 2 Dp1Tyb MEFs v 2 other WT and 2 other Dp1Tyb MEFs. Expressed genes (grey dots) are plotted in chromosomal order. A Loess smoothing curve is superimposed with regions that are apparently up- or down-regulated indicated in red or green.

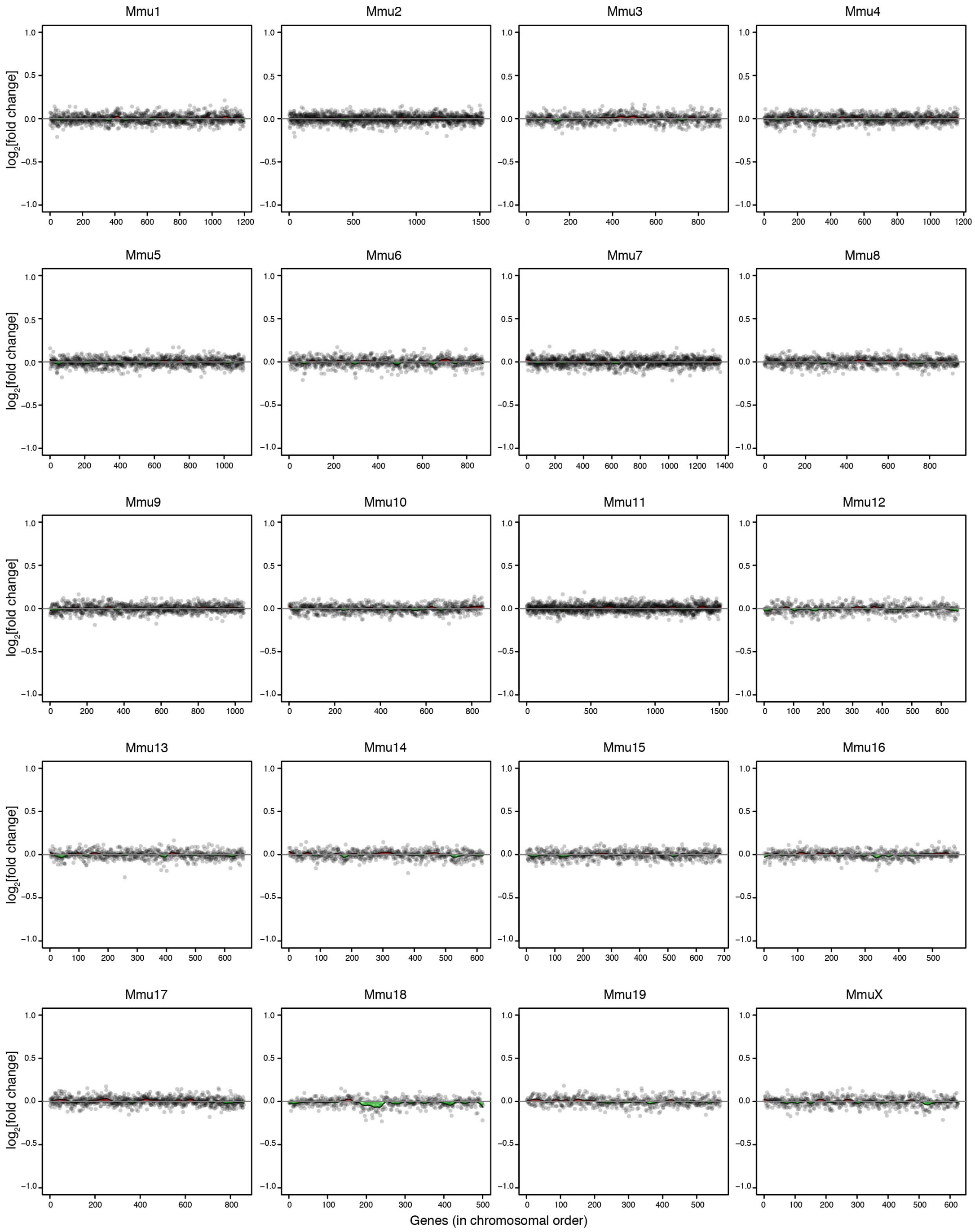

Supplementary Figure 13. Fold changes in gene expression from a no genotype difference comparison of 2 WT and 2 Dp1Tyb hippocampi v 2 other WT and 2 other Dp1Tyb hippocampi. Expressed genes (grey dots) are plotted in chromosomal order. A Loess smoothing curve is superimposed with regions that are apparently up- or down-regulated indicated in red or green.

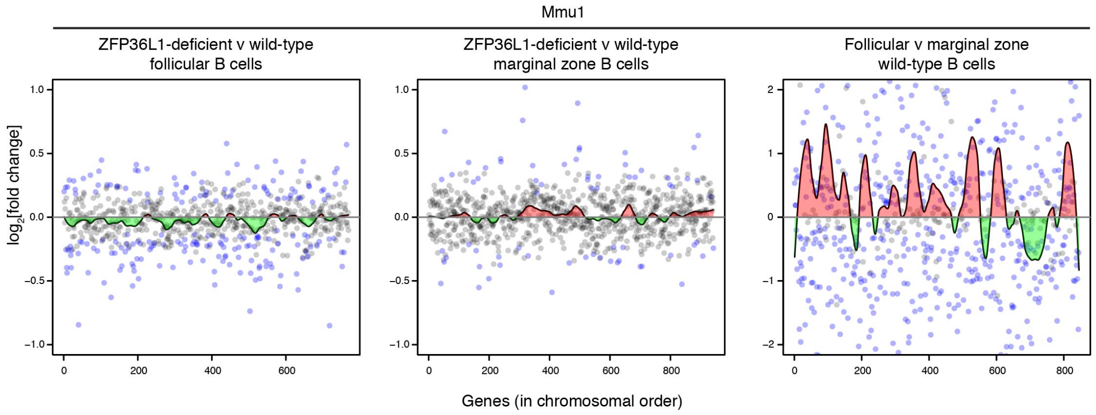

Supplementary Figure 14. Clustered changes in gene expression in ZFP36L1-deficient B cells. Plots show fold change of gene expression in ZFP36L1-deficient v WT follicular (left) or marginal zone (middle) B cells or in follicular v marginal zone WT B cells (right). Expressed genes are plotted in chromosomal order on example chromosome Mmu1. Genes that are significantly differentially expressed are indicated with blue dots, other genes are in grey. A Loess smoothing curve is superimposed with regions that are up- or down-regulated indicated in red or green.

a

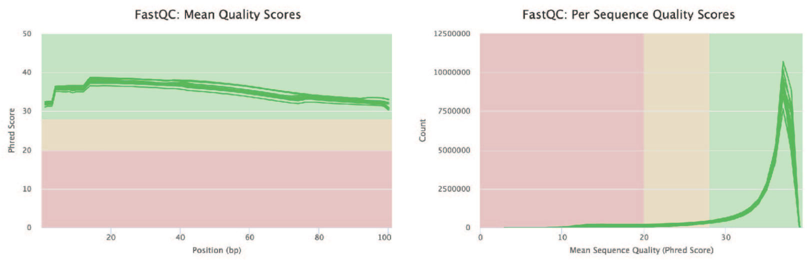

b

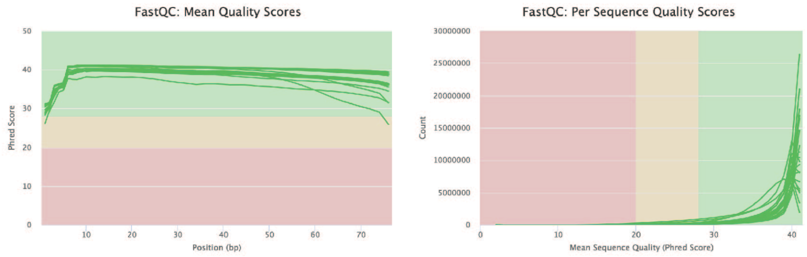

c

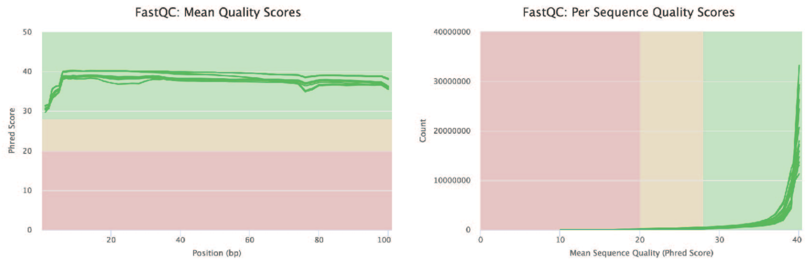

d

Tissue	Sample ID	Genotype	Left Read Input	Left Read Mapped	Right Read Input	Right Read Mapped	Aligned pairs	Unique and concordant aligned pairs	Percentage unique, concordant and aligned pairs
MEF	WT1	WT	34822788	32594609	34822788	31407791	30495854	29497693	84.7%
MEF	WT2	WT	29628026	27579745	29628026	26325226	25568426	24646416	83.2%
MEF	WT3	WT	29351953	27543586	29351953	25585653	24858396	23948918	81.6%
MEF	WT4	WT	30081417	28074510	30081417	27426706	26579175	25628301	85.2%
MEF	DS1	Dp1Tyb	31373357	28883928	31373357	27575048	26762403	25840555	82.4%
MEF	DS2	Dp1Tyb	29074324	27096137	29074324	26236707	25415750	24408831	84.0%
MEF	DS3	Dp1Tyb	30237496	28390681	30237496	27282216	26434949	25531873	84.4%
MEF	DS4	Dp1Tyb	28608722	26693908	28608722	26195098	25364582	24516383	85.7%
MEF	DS5	Dp1Tyb	30371585	28187621	30371585	27221549	26356675	25412260	83.7%
Hippocampus	WT1	WT	39689545	38387652	39689545	37423780	36805799	35160333	88.6%
Hippocampus	WT2	WT	30198192	29250904	30198192	28398579	27958215	26716770	88.5%
Hippocampus	WT3	WT	22639804	21974080	22639804	21336436	20995150	20138060	88.9%
Hippocampus	WT4	WT	40974702	39657860	40974702	38828529	38087667	36507023	89.1%
Hippocampus	WT5	WT	26611304	25865150	26611304	25197222	24808447	23828555	89.5%
Hippocampus	Dp1Tyb1	Dp1Tyb	54473971	52921327	54473971	50853761	50081350	47541562	87.3%
Hippocampus	Dp1Tyb2	Dp1Tyb	35304783	34380965	35304783	33343314	32858997	31411930	89.0%
Hippocampus	Dp1Tyb3	Dp1Tyb	31490822	30535726	31490822	29878090	29383513	28155448	89.4%
Hippocampus	Dp1Tyb4	Dp1Tyb	41956679	39876560	41956679	37337037	36107566	33839611	80.7%
Hippocampus	Dp1Tyb5	Dp1Tyb	27418065	26354075	27418065	25750723	25251537	24313625	88.7%
MEF + ERCC	WT1	WT	44869441	35508000	44869441	33650965	32816193	30440901	67.8%
MEF + ERCC	WT2	WT	48568737	37550241	48568737	35842934	34890030	32300838	66.5%
MEF + ERCC	WT3	WT	42579728	34286823	42579728	32007386	31307939	29293097	68.8%
MEF + ERCC	WT4	WT	42310301	33588000	42310301	32025085	31174607	29125301	68.8%
MEF + ERCC	Dp1_1	Dp1Tyb	49688237	40354237	49688237	38506395	37645316	35103198	70.6%
MEF + ERCC	Dp1_2	Dp1Tyb	33309697	26388175	33309697	25156321	24490900	22266870	66.8%
MEF + ERCC	Dp1_3	Dp1Tyb	39139347	31669737	39139347	30364906	29664182	27616797	70.6%
MEF + ERCC	Dp1_4	Dp1Tyb	36409275	28155570	36409275	26660188	26042928	24321618	66.8%

Supplementary Figure 15. Details of RNAseq experiments. **a, b, c** Mean phred quality scores as a function of position in the sequence and mean quality scores per sequence for RNAseq samples from MEFs (a), hippocampus (b) and MEFs with ERCC spike-ins (c). **d** Table showing data for all RNAseq samples from MEFs, hippocampus and MEFs with ERCC spike-ins listing numbers of left and right reads, mapped reads, and aligned pairs, as well as numbers and percentages of unique, concordant and aligned pairs.
