# Supplementary material for: Gene expression dysregulation domains are not a specific feature of Down syndrome
Source: Nat Commun. 2019 Jun 6;10:2489. doi: 10.1038/s41467-019-10129-9 (PMC6554309; doi:10.1038/s41467-019-10129-9)
Supplement: Supplementary file 3 — Description of Additional Supplementary Files [file 41467_2019_10129_MOESM3_ESM.pdf]

Description of Additional Supplementary Files

File name: Supplementary Data 1

Description: Differential gene expression analysis between Dp1Tyb and WT MEFs. For each gene, the table shows the mean expression, fold change (Dp1Tyb v WT), p-value and p-value adjusted for multiple testing (padj).

File name: Supplementary Data 2

Description: Differential gene expression analysis between Dp1Tyb and WT hippocampus. For each gene, the table shows the mean expression, fold change (Dp1Tyb v WT), p-value and p-value adjusted for multiple testing (padj).
