# Supplementary material for: Gene expression dysregulation domains are not a specific feature of Down syndrome
Source: Nat Commun. 2019 Jun 6;10:2489. doi: 10.1038/s41467-019-10129-9 (PMC6554309; doi:10.1038/s41467-019-10129-9)
Supplement: Supplementary file 6 — Reporting Summary [file 41467_2019_10129_MOESM6_ESM.pdf]

nature research

Corresponding author(s): Victor Tybulewicz
Elizabeth Fisher

Last updated by author(s): Jan 31, 2019

Reporting Summary

Nature Research wishes to improve the reproducibility of the work that we publish. This form provides structure for consistency and transparency in reporting. For further information on Nature Research policies, see Authors & Referees and the Editorial Policy Checklist.

Statistics

For all statistical analyses, confirm that the following items are present in the figure legend, table legend, main text, or Methods section.

n/a	Confirmed	
☐	☒	The exact sample size (*n*) for each experimental group/condition, given as a discrete number and unit of measurement
☐	☒	A statement on whether measurements were taken from distinct samples or whether the same sample was measured repeatedly
☐	☒	The statistical test(s) used AND whether they are one- or two-sided<br>*Only common tests should be described solely by name; describe more complex techniques in the Methods section.*
☒	☐	A description of all covariates tested
☐	☒	A description of any assumptions or corrections, such as tests of normality and adjustment for multiple comparisons
☐	☒	A full description of the statistical parameters including central tendency (e.g. means) or other basic estimates (e.g. regression coefficient) AND variation (e.g. standard deviation) or associated estimates of uncertainty (e.g. confidence intervals)
☐	☒	For null hypothesis testing, the test statistic (e.g. *F*, *t*, *r*) with confidence intervals, effect sizes, degrees of freedom and *P* value noted<br>*Give P values as exact values whenever suitable.*
☒	☐	For Bayesian analysis, information on the choice of priors and Markov chain Monte Carlo settings
☒	☐	For hierarchical and complex designs, identification of the appropriate level for tests and full reporting of outcomes
☐	☒	Estimates of effect sizes (e.g. Cohen's *d*, Pearson's *r*), indicating how they were calculated

Our web collection on statistics for biologists contains articles on many of the points above.

Software and code

Policy information about availability of computer code

Data collection	No software was used to collect data. Data came direct from the sequencing machines.
Data analysis	Software used in the analysis is freely available, except where indicated. The quality of the sequencing data was assessed using FastQC .The adapter sequences were trimmed using TrimGalore! and the reads were mapped to the genome assembly GRCm38 using TopHat (version 2.0.12). Reads mapping to genes were counted using htseq-count. A R/bioconductor package DESeq2 was used for analysis of differentially expressed genes. R/Bioconductor was used to calculate all statistical tests (Loess smoothing function and Pearson correlation) and to visualise the data (MA, correlation and density plots). Custom scripts were used to calculate the flip numbers and energy metrics, and to align boundaries of GEDDs and TADs with genomic elements. Custom scripts have been uploaded to GitHub.

For manuscripts utilizing custom algorithms or software that are central to the research but not yet described in published literature, software must be made available to editors/reviewers. We strongly encourage code deposition in a community repository (e.g. GitHub). See the Nature Research guidelines for submitting code & software for further information.

Data

Policy information about availability of data

All manuscripts must include a data availability statement. This statement should provide the following information, where applicable:

- Accession codes, unique identifiers, or web links for publicly available datasets
- A list of figures that have associated raw data
- A description of any restrictions on data availability

All RNAseq data has been deposited in the Gene Expression Omnibus, accession number GSE109295.

Field-specific reporting

Please select the one below that is the best fit for your research. If you are not sure, read the appropriate sections before making your selection.

☒ Life sciences ☐ Behavioural & social sciences ☐ Ecological, evolutionary & environmental sciences

For a reference copy of the document with all sections, see nature.com/documents/nr-reporting-summary-flat.pdf

Life sciences study design

All studies must disclose on these points even when the disclosure is negative.

Sample size	No sample size calculation was performed ahead of data acquisition. A minimum of 4 independent replicates were used of each genotype for both MEFs and hippocampus, a number that previous experience has shown is sufficient for statistical analysis of differential gene expression.
Data exclusions	No data was excluded from the analyses.
Replication	The RNAseq of MEFs was carried out on 4 WT and 5 Dp1Tyb MEF independent cultures, each derived from a different embryo. RNAseq of hippocampus was carried out on 5 WT and 5 Dp1Tyb hippocampus each isolated from a different animal. Thus each RNA sample that was sequenced is an in independent replicate.
Randomization	Samples for RNAseq were allocated into groups according to genotype (WT or Dp1Tyb).
Blinding	No blinding was performed in this study by the investigators carrying out the purification of RNA from MEFs or hippocampus. However the library preparation and sequencing was carried out by individuals blind to the genotype of the samples.

Reporting for specific materials, systems and methods

We require information from authors about some types of materials, experimental systems and methods used in many studies. Here, indicate whether each material, system or method listed is relevant to your study. If you are not sure if a list item applies to your research, read the appropriate section before selecting a response.

Materials & experimental systems

n/a	Involved in the study	
☒	☐	Antibodies
☒	☐	Eukaryotic cell lines
☒	☐	Palaeontology
☐	☒	Animals and other organisms
☒	☐	Human research participants
☒	☐	Clinical data

Methods

n/a	Involved in the study	
☒	☐	ChIP-seq
☒	☐	Flow cytometry
☒	☐	MRI-based neuroimaging

Animals and other organisms

Policy information about studies involving animals; ARRIVE guidelines recommended for reporting animal research

Laboratory animals	The study used C57BL/6J.129P2-Dp(16Lipi-Zbtb21)1TybEmcf/Nimr (Dp1Tyb) mice, which were bred against C57BL/6JNimr mice to generate Dp1Tyb and WT mice. All studies used paired littermate Dp1Tyb and WT mice. The MEFs were isolated from embryos at E14.5, whose gender was not determined. The hippocampus samples came from male mice aged 18.5-19 weeks.
Wild animals	No wild animals were used in this study.
Field-collected samples	The study did not involve any samples collected from the field.
Ethics oversight	All animal work was carried out under Project Licences granted by the UK Home Office.

Note that full information on the approval of the study protocol must also be provided in the manuscript.
